# Inflammation of the Gums

**Published:** 1870-10

**Authors:** J. P. H. Brown


AMERICAN JOURNAL

OF

### DENTAL SCIENCE.

Vol. IV. THIRD SERIES--OCTOBER, 1870. No. 6.

### ARTICLE I.

### *Inflammation of the Gums.*

By J. P. H. Brown, D. D. S.

**Read before the Georgia State Dental Society.**

There is no portion of the mucous membrane of the mouth of which the dentist is called upon to treat, that taxes his skill and remedial resources more than inflammation of the gums.

As they are connected with the alveolo-dental periosteum and alveolar processes, and immediately contiguous to the teeth, they are at all times liable to be affected by any morbid action existing in these parts, moreover, being composed of thick, dense mucous membrane, which is the same tissue that lines the mouth, lungs, alimentary canal, and in fact, all the cavities and hollow organs which communicate externally by different apertures on the skin, and being endowed with a high degree of vascularity, it may well be supposed that their condition of health is also influenced to a very great extent by certain constitutional tendencies.

It is impossible in a short paper to discuss all the pathological conditions of the gums. This would require a volume. I shall only consider those phases of inflammation usually known by the terms *acute* and *chronic*, and which constantly occur in a dental practice. I lay no claim to any new or wonderful discovery, or infallible mode of treatment, but will describe the course of practice which has been the most successful in my own hands.

The physician has more to do with acute inflammation of the gums than the dentist. Active inflammation of the gums does not always commence within their structure, but is sometimes the result of lesion of the adjacent parts; as for instance in *follicular stomatitis*, a disease confined mostly to young children. Here the disease commences in the mucous follicles of the tongue, fauces and inside of the mouth and the gums become affected sympathetically.

The only forms of acute inflammation of the gums that come more especially within the practice of the dentist are *ulcerative stomatitis* and *mercurial action.*

In *ulcerative stomatitis* the gums are attacked first, and the ulceration often progresses to such an extent as to lay bare the roots of the teeth. Dr. Tanner, when speaking of this disease in his "*Principles of Medicine*," observes: "On looking into the mouth we shall see that the gums are swollen, red or violet colored, readily bleeding to the touch, and covered with a layer of pulpy grayish matter. If the disease be allowed to creep on unchecked, the gums will get destroyed by the ulceration, and the teeth become exposed and loosened until they fall out. The morbid action also spreads to the inside of the cheeks, which become covered with irregular sloughing ulcerations; and the tongue assumes a swollen and sodden appearance. Ulcerative stomatitis is not uncommon; it occurs from the most part in weakly children who have been badly nourished, and exposed to cold and damp.

"The treatment of this disease is not difficult, inasmuch as we possess in the chlorate of potash a remedy which may almost be deemed a specific. Five grains of this salt may be given every four or six hours to an infant one year old, in a little sugar and water. When the ulcerations have healed, bark or quinine should be administered."

Local treatment is regarded in this disease of secondary importance. Nitrate of silver may be applied to the ulcers, which will change their character and relieve the excessive tenderness. A weak solution of carbolic acid applied as a wash will suppress the offensive odor. All necrosed teeth and roots which are a source of irritation should be extracted, and all collections of tartar removed.

In acute inflammation of the gums caused by mercury, the first appearance of the trouble is indicated by a peculiar tenderness and swelling of the gums, and by an increased secretion of mucus and glandular fluids into the mouth. These symptoms rapidly increase, and are accompanied by a very disagreeable odor, and a metallic taste in the mouth. If the use of the mercurial preparations is continued, these symptoms become very much aggravated. The gums become greatly swollen and covered with ulcers, the salivary glands become greatly affected and pour out an excessive amount of secretion, and extensive sloughing of the soft parts and bones sometimes occurs. In the treatment of mercurial salivation great benefit has been derived from the use of chlorate of potash. Mild aperients, combined with a well regulated diet and fresh air, must be resorted to. The local applications should consist of astringent and detergent washes.

*Chronic inflammation* of the gums is a disease for the relief of which the assistance of the dentist is generally sought, and to a description of which I shall confine the remaining portion of this paper.

In this form of inflammation, the gums are usually of a livid red appearance, soft and spongy, and bleed upon the slightest touch. Their surface when closely examined is found to be minutely nodulated, and the secretion of epithelium increased; the papillæ are abnormally prominent, and the substance of the gum around and between the teeth is thickened and enlarged. In some cases there is so much enlargement of gum that crowns of the teeth may be nearly or entirely covered, rendering the process of mastication nearly impossible.

In the treatment of this disease it is of the utmost importance to get at the cause of the difficulty. If the cause is local, that is, arising from the presence of diseased teeth, roots, tartar, &c. these irritants should at once be removed. If the gums are very much enlarged with great prominences between the teeth, the enlarged portion should be cut away with a lancet or a sharp scaler. Scarification will do good at all stages of the disease. After the bleeding has somewhat subsided, the inflamed surface should be touched with a pointed stick of nitrate of silver. Though I have found better results to follow (and much prefer) an application of a mixture of equal parts of creosote and tincture of iodine applied with a camel's hair brush. It is always best to alternate these caustic applications with an astringent and alterative wash, such as a decoction of red-oak bark, tincture of arnica, or a weak solution of sulphite of soda or chlorate of potash.

There are many cases where the trouble is more constitutional than local. In these cases the gums seem to be the point where the hidden lurking poison in the system shows itself. This poison may be mercury, scrofula, scorbutus, or, perchance, some syphilitic virus that has been poisoning, as it were, under ground for several generations. It may also result from disordered digestion, or from a general weakness of the system. In all such cases local applications will prove of little benefit unless the system be toned up and invigorated by general treatment.

There is another form of chronic inflammation of the gums where the disease is accompanied by a wasting away of the alveolar processes, and the gradual recession of the gums from the teeth. This form is the most difficult to treat, and often resists most obstinately all the treatment and means the dentist can bring to bear against it. It usually commences in the gums around a single tooth, or it may attack the gums of the whole denture.

Those persons with a scorbutic or rheumatic diathesis are more subject to it than others. We find the tendency to this disease to run in families. I know of a mother who lost all her teeth by this disease, and three of her children who have inherited her constitutional predisposition have the same disease in their gums. But this is not all. In the mother's case the disease commenced around the left lower central incisor, and in her children the disease made its appearance around the identical tooth.

Tartar and dead teeth are not the sole cause of this disease as many suppose. They only irritate and keep alive the morbid susceptibility (if I may so speak) existing in the gums. Furthermore, the tartar that is deposited deeply down toward the apex of the root is no salivary deposit at all, but a formation or precipitate from the secretion thrown out by the diseased gums. This tartar has a dark greenish appearance—sometimes tinged with yellow—and presents a nodular formation. It has altogether a different appearance from salivary calculus.

When the disease first makes its appearance in the gum around a single tooth, the gum often at first presents a sort of ulcerous look. It is a bright red, but soon changes to a more livid color. The alveolo-dental periosteum also being implicated in the morbid action, the edge of the alveolus disappears, the gum sinks down, the tooth becomes loose, and by slow degrees loses its implantation and falls out. Tooth after tooth in this manner may be lost.

The predisposing cause of this form of chronic inflammation of the gums is unquestionably a constitutional susceptibility of the gums to morbid action. The exciting causes are necrosed teeth and roots, tartar and foreign deposits around the teeth irregularity and pressure of the teeth, badly fitting plates, and the effects of mercury. An artificial denture that does not fit when applied to a mouth with these irritable gums is apt to keep up the disease, and there may be a slow wasting of the alveolar ridge until it is very much absorbed. The gums over this absorbed portion are thick and soft—feeling like a mass of thick flesh.

The great majority of patients suffering with this disease not only require judicious local treatment but also general treatment. In fact, without the latter the former will prove of little benefit. Every means should be used calculated to correct, improve and invigorate the general system. The diet should be nutritious ; and embrace more fruit and vegetables and less animal. All salted meats should be eschewed. The most difficult cases to cure we always find among those whose habits of life are not correct—who drink and dissipate. The local treatment should be pretty much the same as that for the common form of chronic inflammation. But it must faithfully be kept up if we expect success. The creosote and iodine mixture alternated in its use with sulphite of soda, tannic acid, &c., can not be too highly recommended. Every particle of tartar, all dead roots, and loose teeth that will not tighten and are of no service should be removed. In rare cases it may be very desirable to retain some teeth that are very loose. This can sometimes be satisfactorily done by wiring them to those that are firm. Loose teeth have been retained in this manner so as to be servicable for many years.

---

